# The Relative Importance of Language in Guiding Social Preferences Through Development

**DOI:** 10.3389/fpsyg.2016.01645

**Published:** 2016-10-20

**Authors:** Rana Esseily, Eszter Somogyi, Bahia Guellai

**Affiliations:** Laboratoire Ethologie, Cognition, Développement, Université Paris Ouest Nanterre la DéfenseNanterre, France

**Keywords:** language, categorization, social preferences, socio-cognitive development, social behavior

## Abstract

In this paper, we review evidence from infants, toddlers, and preschoolers to tackle the question of how individuals orient preferences and actions toward social partners and how these preferences change over development. We aim at emphasizing the importance of language in guiding categorization relatively to other cues such as age, race and gender. We discuss the importance of language as part of a communication system that orients infants and older children’s attention toward relevant information in their environment and toward affiliated social partners who are potential sources of knowledge. We argue that other cues (visually perceptible features) are less reliable in informing individuals whether others share a common knowledge and whether they can be source of information.

## Introduction

For efficient interactions, we need to form cognitive representations of our social partners and of human groups in general. Several studies describe the social cues and categories that influence adults’ everyday interactions and have important downstream consequences for how we construe others (Tajfel et al., 1971; Stangor et al., 1992). There is a growing body of literature on how infants and children process interactive situations and the cues that orient their social preferences and behaviors. Some cues are well documented and show a strong bias for categorization and for guiding infants’ social behavior, namely gender, age and ethnic origin. Language on the other hand, has received interest only more recently despite the fact that individuals are exposed to their native language already in their mothers’ womb, that language is shared by communities, and is a vector for cultural learning. The aim of the present paper is to review evidence showing that language is a special cue as important, if not more important, than gender, age or ethnic origin in guiding social categorization and preferences. The hypothesis we propose is that language, unlike other cues, is a marker for cultural affiliation where social partners share the same norms and are knowledgeable. Through childhood, it is important for developing human beings to pay attention to cues that guide them toward potential sources of information and learning. For learning, choosing a native speaker as a social partner is a relevant matter, however, choosing an individual upon his ethnic origin may not be reliable. The relative importance of these categorizations can also change with age as older children learn more about each of these categories and build a hierarchy model of their world. We shall first review studies that focused on gender, race and ethnic origin on the one hand and language on the other hand in infants, toddlers, and preschoolers. Then we shall proceed with studies that weighed the relative importance of these cues.

## Gender, Age, Race and Ethnic Origin

One of the most salient and robust cues in early life along which we can divide our social world is gender. Very early in life, infants already show preferences according to the gender of the photograph presented to them: 3- and 4-month-old infants prefer looking at female faces rather than male faces (Quinn et al., 2002). Knowledge of gender categories increases during the second year of postnatal life. By 18 months, girls are able to match gender labels with appropriate faces (Poulin-Dubois et al., 1998) and infants’ tendency to categorize dolls according to gender increases sharply between 18 and 22 months of age (Johnston et al., 2001). At 18 months infants also start to show awareness of gender-associated toy stereotypes by looking longer at faces that match the gender stereotyping of a previously presented toy (Serbin et al., 2001) and by showing specific patterns of sequential touching of gender-typed toys associated with their own sex (Levy, 1999). At the beginning of their second year, toddlers show awareness of the typical activities of men and women by looking longer at surprising, stereotype-inconsistent photographs than at stereotype-consistent ones (Poulin-Dubois et al., 2002) and tend to select a ‘sex appropriate’ doll when imitating a gender stereotyped action (Serbin et al., 2001).

Age also emerges as an early cue and by 6 months of age, infants already categorize faces from different age groups as they prefer looking at images of same age peers rather than images of older infants (Sanefuji et al., 2006). Age is an important indicator of an observed person’s knowledgeability, which further contributes to guiding infants’ attention. Thus, Zmyj et al. (2012) found that 12-month-olds (and also younger infants but less reliably) preferred to observe older children. The authors argue that older children provide both a level of similarity as well as increased competence that creates a zone of proximal development (Vygotsky, 1978) for the younger, which possibly prefer to observe older peers in order to learn from them. Indeed, in Vygotsky’s theory (Vygotsky, 1978), more capable peers who guide children in performing an activity create a zone of proximal development, which allows children to perform activities at a higher level than the level at which they could perform independently.

As to ethnic origin, it has been shown that preference for own-race versus other-race faces appears at 3-months of age (Quinn et al., 2002; Kelly et al., 2005; Bar-Haim et al., 2006). However, Social preferences based on ethnic origin emerge only between 2.5 and 5 years of age (Kinzler and Spelke, 2011), which is later than gender-, age- and language-based preferences. Considerable amount of research has examined the development of children’s attitudes toward members of their own and other ethnic groups. These studies have revealed that children, and especially those living in multi-ethnic communities, can categorize people based on physical cues (e.g., skin color) and by around 4 years of age their ethnic awareness enables them to distinguish between members of different ethnic groups. By 6–7 years of age, children identify themselves with their own ethnic groups, exhibiting preference and positivity toward members of their own groups and negativity toward members of other ethnic groups (see reviews by: Aboud, 1988; Nesdale, 2001). Thus, race while certainly important for older children may not be as important for young children early in development. Possibly because in early development the ethnic origin of an individual does not convey any information to the infant other than his perceptible properties, whereas age can be an indicator for expertise, competence, reliance and authority and gender can give reliable information about possible areas of interest. Thus, infants and children may have expectations about shared knowledge with someone sharing or not the same expertise (age) and interest (gender) unlike someone only sharing the same color.

## Language as a Marker Guiding Social Behavior

Language provides a wide range of information about people such as their geographic origin, social status, gender, and ethnic group (Labov, 1976, 2001). The reason why language can be considered as a strong cue in development is threefold: (1) it drives social preferences early in development, (2) it is a social marker for affiliation and social interaction and (3) language is a vector for social and cultural learning. In this section, we will review evidence for each of these functions.

Language is already an important cue in guiding infants’ early social preferences, and more particularly in-group preferences. Recent evidence suggests that newborns prefer to look at a person who previously spoke to them than at someone who was silent (Coulon et al., 2011; Guellai and Streri, 2011). Soon after birth, and throughout early infancy, young infants prefer listening to their native language rather than to a foreign one (Mehler et al., 1988). They can also discriminate among different languages based on rhythmic or phonological cues (Mehler et al., 1988; Bosch and Sebastian-Galles, 1997; Nazzi et al., 1998; Best and McRoberts, 2003; Kuhl et al., 2006; Weikum et al., 2007). Beyond these early achievements, language can guide infants’ social preferences: infants as young as 6-months prefer looking at the video of a woman who previously talked to them in their native language with a native accent (i.e., American English), than at a woman who previously spoke in a foreign language (i.e., Spanish) (Kinzler et al., 2007). At 7-months, they prefer listening to a tune that had been introduced by a native speaker compared to a tune introduced by a foreign speaker (Soley and Sebastián-Gallés, 2015). At 10 months they preferentially choose toys offered by a native speaker over a toy offered by a non-native one (Kinzler et al., 2012) and at 12 months they select food that was first tasted by a native rather than a non-native speaker (Shutts et al., 2009). Dialect may also be a reliable and more precise cue to social preferences because it provides information about an individual’s social and ethnic identity. In a recent study, Okumura et al. (2014) showed that 9- and 12-month-old infants preferentially touched a toy offered by a native-dialect speaker compared to a toy offered by a non-familiar dialect speaker. These findings clearly show the importance of infants’ social and linguistic environment in the early development of social preferences.

Other studies put forward the role of language in marking affiliation and in guiding social interactions (Kinzler et al., 2010, 2011; Kinzler and Dautel, 2012; Howard et al., 2015). Most children choose faces paired with native-accented voices as friends and they consider them nicer compared to faces paired with non-native accented voices (Kinzler and DeJesus, 2013) and this is also true for bilinguals (Souza et al., 2013). In a recent study (Liberman et al., 2016), the affiliative function of language was directly tested in 9-month-old infants. Infants saw a video of two actors who either spoke the same language (English–English or Spanish–Spanish) or different languages (English–Spanish). Then, in the test phase, infants saw videos of the same actors either showing affiliation (waving and smiling to each other) or disengagement (turning their back to each other). Infants in this study expected affiliation behavior when the actors spoke the same language and were surprised when the actors disengaged. When the actors spoke different languages, infants were surprised when they exhibited affiliation behavior compared to disengagement. Taken together, these studies show that infants use language as a marker for affiliation and for social interactions.

Furthermore, there is evidence that language also constitutes a strong cue for learning by pointing out the knowledgeability of a social partner. Oláh et al. (2014) investigated how the language a model speaks (foreign or native) is associated with the conventionality of this model’s tool use habits (conventional or unconventional). They found that 2-year-olds associated a foreign language to the model if he had previously performed goal-directed actions in a non-conventional way (e.g., comb hair with fork), but formed an association between the foreign language and another person if previously the model had been seen to act in a conventional way (eat with fork), making it unlikely that he was the source of the foreign language utterance. This shows that language conveys as well as affiliation, social norms and cultural information about the knowledge members of a community share and thus potential sources of learning. Few studies investigated how language can be a vector for learning. These few studies show that 14-month-olds infants (Buttelmann et al., 2013) and preschool children (Kinzler et al., 2011; Howard et al., 2015) selectively imitate a novel action demonstrated by a native-accented speaker compared to a non-native accented speaker. However, this preference is modulated by other factors such as accuracy: even though preschoolers prefer labels provided by native speakers compared to non-native speakers, they override their preference when the native speakers are not accurate in labeling familiar words for example (Corriveau et al., 2013). Thus, accuracy but also morality has been shown to influence the linguistic in-group bias (Kinzler and DeJesus, 2013; Hetherington et al., 2014).

## How Do Children Weigh Social Cues?

The studies mentioned above show both the flexibility with which children divide their social world, as well as the importance of gender, race, age and language in guiding children’s social preferences and behavior toward others. Most of these studies, however, target children’s preferences between modalities of the same social category (between the two genders or two races for instance), and do not directly compare the relative importance of categories when compared to one another. Yet social partners stand at the crossroads of multiple social categories, which are thus interdependent, meaning that the impact of each social cue is weighted differently according to the context. We can note that relatively few studies have investigated how intersectionality unfolds early in development (for a review see Kinzler et al., 2010). Thus, in addition to examining social category emergence, an important direction for future research is to investigate priorities in children’s social categories by directly comparing the influence of more than one category and investigating how cues depend from one another with the same method and population of children.

What determines the priority of one category over another? For adults, the influence of age, gender, and race has been attributed to each category’s visual salience (Fiske, 1998). It is possible that children’s social category formation is also largely reliant on visual observations of properties that differ among individuals, since these factors are noticeable with minimal effort. Indeed, children demonstrate in-group biases based on minimal groupings for “blue” and “yellow” groups created by labeling and a visual cue to group membership (different colored t-shirts that are randomly assigned), but not in the absence of supporting visible distinctions (Bigler et al., 1997). Beyond visual salience, however, findings from evolutionary psychology show that evolution by natural selection may have favored attention to certain social categories over others – for example, gender over race—and that this relative weighting is continually visible in adulthood (Kurzban and Leary, 2001).

Studies comparing different categories confirm this evolutionary perspective and show that encoding social categories is not automatic. Even though, race appears to be a more salient cue for directing visual preference and can override gender at 3 months of age (Kelly et al., 2005, 2007; Quinn et al., 2008), somewhat later in childhood though and also in adults (Kurzban and Leary, 2001), it becomes a less privileged marker of social category membership than gender or age. For example, Weisman et al. (2015) showed that 3- to 6-years old children showed a preference bias toward both gender and race but they were more likely to learn facts about children of different gender than children of the same gender and equally likely to confuse targets within and across racial groups. This clearly shows that race is a less fundamental social category compared to gender and does not constitute an important cue for learning. Again, when 3-year-old white preschoolers were asked to choose between objects or activities that were presented by unfamiliar people who differed in gender, race (white, black), or age (child, adult), gender and age were more robust guides to children’s preferences than race (Shutts et al., 2010). In a further study (Rhodes and Gelman, 2009) in which 5-year-olds were prompted to reason about the categorization of others, children viewed gender as a naturalized category that is objectively determined. In contrast, race was seen as flexibility determined, similarly to how children reason about artifacts. These findings indicate that in preschoolers, gender and age are both reliable categories and used more robustly and consistently than race.

Next to gender and age, language also emerges as hierarchically superior to other characteristics along which infants, toddlers and preschoolers form judgments about a person. Given the importance of language in guiding infants’ and children’s social interactions, more studies weighing language and other social categories are needed to fully understand the relative importance of language. There is some evidence that language (more particularly accent) takes priority over other cues such as race at 5 years of age (Kinzler et al., 2009, 2010; Kinzler and Dautel, 2012). For instance, children choose native-accented speakers as friends, even when they are of a different race (Kinzler et al., 2009), which shows that children are sensitive to cultural markers beyond physical similarities.

## General Discussion

The aim of this review was to answer two questions: (i) how do infants, toddlers and preschoolers categorize their potential social partners and orient their social preferences and (ii) what is the relative importance of language in guiding these behaviors? The studies reviewed clearly show that from early on and as young as 3 months of age, infants categorize individuals on the basis of physical and linguistic characteristics. They also show that infants’ and children’s social behaviors are deeply influenced by group identity and membership. Studies in older children show that children’s preferences are not based on experience and familiarity only but rather on sharing cultural norms and knowledge with members of a given group. They also show that group memberships are not immutable and behaviors toward in-groups and out-groups can be modulated by other contextual information such as morality or accuracy.

Children can also prioritize available cues: indeed, from birth, infants are oriented toward elements that make sense in their environment and from which they can learn something. Their curiosity and internal motivation make them explore their environment with the goal of making new discoveries. In their everyday exploration, infants encounter social partners who also have the power to transmit some knowledge about the world. Characteristics of these partners are crucial to pay attention to because they indicate whether the partner is knowledgeable or not. Age and gender become rapidly robust cues that refer to expertise and competence while race is not related to partner’s ability to teach something new. Language on the other hand is special and maybe even more important than other cues because as seen above it guides early social preferences and it is both a marker for affiliation and for knowledgeability as it is shared between people of a same community and it vehicles a multitude of new information. Further studies weighing language and other social categories are required to better understand the relative importance of language.

Another question that needs to be addressed to capture the full picture is the universality of some of these markers and specifically language as a marker for group affiliation. Indeed if language is important in guiding children’s behavior and overrides other cues such as race, then it is probably a common cue detected very early in life helping infants to orient their attention toward members of their own community with whom knowledge may be shared.

## Author Contributions

All authors listed, have made substantial, direct and intellectual contribution to the work, and approved it for publication.

## Conflict of Interest Statement

The authors declare that the research was conducted in the absence of any commercial or financial relationships that could be construed as a potential conflict of interest.
